# Growth of etiolated barley plants in weak static and 50 Hz electromagnetic fields tuned to calcium ion cyclotron resonance

**DOI:** 10.1186/1477-044X-4-1

**Published:** 2006-02-03

**Authors:** Alexander Pazur, Valentina Rassadina, Jörg Dandler, Jutta Zoller

**Affiliations:** 1 Department Biologie I Universität München – Bereich Botanik, Menzingerstr. 67, D-80638 München, Germany; 2 Institute of Biophysics and Cell Engineering, National Academy of Sciences of Belarus Academicheskaya 27, Minsk 220072 Belarus, Germany

## Abstract

**Background:**

The effects of weak magnetic and electromagnetic fields in biology have been intensively studied on animals, microorganisms and humans, but comparably less on plants. Perception mechanisms were attributed originally to ferrimagnetism, but later discoveries required additional explanations like the "radical pair mechanism" and the "Ion cyclotron resonance" (ICR), primarily considered by Liboff. The latter predicts effects by small ions involved in biological processes, that occur in definite frequency- and intensity ranges ("windows") of simultaneously impacting magnetic and electromagnetic fields related by a linear equation, which meanwhile is proven by a number of *in vivo *and *in vitro *experiments.

**Methods:**

Barley seedlings (*Hordeum vulgare*, L. var. Steffi) were grown in the dark for 5 and 6 days under static magnetic and 50 Hz electromagnetic fields matching the ICR conditions of Ca^2+^. Control cultures were grown under normal geomagnetic conditions, not matching this ICR. Morphology, pigmentation and long-term development of the adult plants were subsequently investigated.

**Results:**

The shoots of plants exposed to Ca^2+^-ICR exposed grew 15–20% shorter compared to the controls, the plant weight was 10–12% lower, and they had longer coleoptiles that were adhering stronger to the primary leaf tissue. The total pigment contents of protochlorophyllide (PChlide) and carotenoids were significantly decreased. The rate of PChlide regeneration after light irradiation was reduced for the Ca^2+^-ICR exposed plants, also the *Shibata *shift was slightly delayed. Even a longer subsequent natural growing phase without any additional fields could only partially eliminate these effects: the plants initially exposed to Ca^2+^-ICR were still significantly shorter and had a lower chlorophyll (a+b) content compared to the controls. A continued cultivation and observation of the adult plants under natural conditions without any artificial electromagnetic fields showed a retardation of the originally Ca^2+^-ICR exposed plants compared to control cultures lasting several weeks, with an increased tendency for dehydration.

**Conclusion:**

A direct influence of the applied MF and EMF is discussed affecting Ca^2+ ^levels via the ICR mechanism. It influences the available Ca^2+ ^and thereby regulatory processes. Theoretical considerations on molecular level focus on ionic interactions with water related to models using quantum electrodynamics.

## Background

Weak magnetic fields (MF) and low frequency electromagnetic fields (EMF) were ubiquitous environmental factors on the earth at every time. The former mainly originates from the geomagnetic field, the latter from complex interactions of weather phenomena and impacting cosmic particles in the atmosphere, which result in electromagnetic sphere resonances around the globe, e.g. the *Schumann *resonance [1]. These natural EMF are widely determined by the solar activity, e.g. the sunspot maximum recurring approximately every 11 years, they possibly even modulate our climate [2].

More recent contributions to the man made EMF are related to electrification and wireless communication in large regions of the world. They include particular frequencies of 50 Hz (resp. 60 Hz for Northern America). Projects were undertaken to elucidate potential risks of these EMF for human health, which include a WHO study [3]. Later research increasingly focused on the molecular level. Redistributions of membrane proteins by 50 Hz EMF is reported by Bersani et al. [4]. The expression of heat shock genes in human cells in response to extremely low-frequency EMF of 10 mT could be shown by Tokalov and Gutzeit [5]. Blackman et al. [6] found an enhanced efflux of calcium ions from chicken brain tissue in the presence of 50 Hz modulated radio frequencies. Reports of the modulation of free radical generation (Rollwitz et al. [7]) were related to a possible enhanced cancer risk. Investigations on 60 Hz fields indicated, among others, an increase of DNA strand breaks [8] and a suppressed melatonin production [9]. EMF effects on plants are less studied, but may contribute equally to the understanding of the underlying basic mechanisms. Davies [10] reported an increase in weight and size for radish with EMF of 60 Hz and 40 μT. Frequency dependent changes in membrane permeability were reported for barley seeds by Khizhenkov [11]. An increased cell division correlated with several amplitude optima between 50 and 200 μT of a 7.8 Hz EMF in three species of green algae [12].

Several mechanisms have been proposed for the observed effects of MF and EMF. Two of them, the orientation of ferrimagnetic particles and the modulation of radical-pair reactions [13], have been studied in considerable detail e.g. on the orientation system of migratory birds [14] and other animals. They are theoretically well understood. There exist however an increasing number of investigations showing EMF effects on systems without proven ferrimagnetism, and at field strengths well below the radical pair mechanism. Such a mechanism needs overcoming the environmental thermal noise, which is energetically several orders greater ("*kT *problem") than the excitation energy enforced by EMF.

Some of these effects have been related to ion cyclotron resonance (ICR) of ions dissolved in the aqueous phase. It occurs for predictable superposition of a static MF with field strength **B**_***DC ***_and, as a rule, a EMF with a distinctly weaker amplitude **B**_***AC***_. This mechanism is related to the equation:



where **B**_***DC ***_is the flux density of the applied MF, *f *the frequency of the superimposed EMF,*m *the mass and *q *the charge of the ions involved. Liboff [15] suggested that magnetic fields can interact in a resonant manner with endogenous AC electric fields in biological systems. Binhi [16] reviewed the mechanisms of magnetobiological effects, and tried to estimate the sensitivities and involved molecular topologies. Adair [17] questioned altered transition rates of excited ions by weak EMF for the ICR effect, while an involvement of quantum electrodynamic properties of water is suggested by Del Giudice et al. [18]. The germination rate of *Raphanus sativus *was affected when conditions were applied for Ca^2+^, K^+ ^and Mg^2+ ^[19]. It is noteworthy that ICR conditions for many biologically important ions can be met by combinations of the local geomagnetic field and man-made electromagnetic fields, including the power line frequencies of 50 or 60 Hz.

Ca^2+ ^ions are in particular essential regulatory components of all organisms. Being a second messenger, Ca^2+ ^is involved in regulation at all stages of plant growth and development, including growth and differentiation, photomorphogenesis and embryogenesis, the self-incompatibility responses in pollen-pistil interactions, perception of symbiotic signals, hypersensitive responses induced by pathogens and elicitors, gravitropism and phototropism, assembling and disassembling of cytoskeleton elements, perception of red and blue light, cyclosis, and movement of stomatal cells [20].

Accordingly Ca^2+ ^is the most investigated ion for the ICR effect. Ca^2+ ^incorporation into human lymphocytes was studied as a function of EMF frequencies up to 30 Hz. Sharp resonances were taken as evidence, that ICR links electromagnetic fields and cell transport processes [21].

This work tries to elucidate EMF effects on early plant development, which occur under conditions tuned to the ICR of Ca^2+^. Besides morphological data, the content of the photosynthetic pigments, as well as the changes in absorption spectra at 650 – 684 nm of etiolated leaves after short illumination were determined by *in vivo *spectroscopy. The latter allows to investigate *in vivo *two processes in greening plants: The regeneration of photoactive protochlorophyllide a 650 (PChlide650) seen at 650 nm and the Shibata shift, which is the shift of chlorophyllide (Chlide) absorption maximum from 684 to 672 nm during about 30 min after light exposure. The molar amount of Chlide undergoing the Shibata shift is, at any time, identical with the amount of Chlide being esterified [22], it is therefore possible to consider kinetics of blue shift as the kinetics of Chlide esterification. The Shibata shift, which is the shift of chlorophyllide (Chlide) absorption maximum from 684 to 672 nm during about 30 min after light exposure, and the longer lasting regeneration of protochlorophyllide *a *650 (PChlide650) seen at 650 nm are investigated in detail by [22,23].

## Methods

### Plant growing

Barley seeds (*Hordeum vulgare*, L. var. Steffi) were grown in quadratic (9.5·9.5 cm, height 11 cm) plexiglass boxes. The lid had spacers on the edge and enabled an uniform air circulation to the interior space from all sides, with a total opening of 28 mm^2^. Lower parts and lids of the boxes were covered with black, lighttight plastic foil for reaching a completely dark inside. Every box contained 10 g Vermiculite, to which 70 ml deionized water (50 ± 10 μS) was added. 5 ± 0.05 g Barley, corresponding to 105 ± 1 seeds were evenly distributed on the moist Vermiculite evenly distributed as possible. The soaking seeds were covered with additional 2 g Vermiculite. The boxes were closed and incubated for 5 days in the dark at a temperature of 21 ± 0.5°C. They were weighed daily until harvest. Afterwards, part of the seedlings were used for immediate investigations, another part was grown additional 24 hours in the darkness or at normalized illumination for special tests. The remaining individuals were fertilized with a nutrient solution after *Knob *[24] and subsequently exposed to daylight for greening, these plants were repotted on soil and kept in a greenhouse for further observation.

### Electromagnetic field exposure

**Figure 1:**
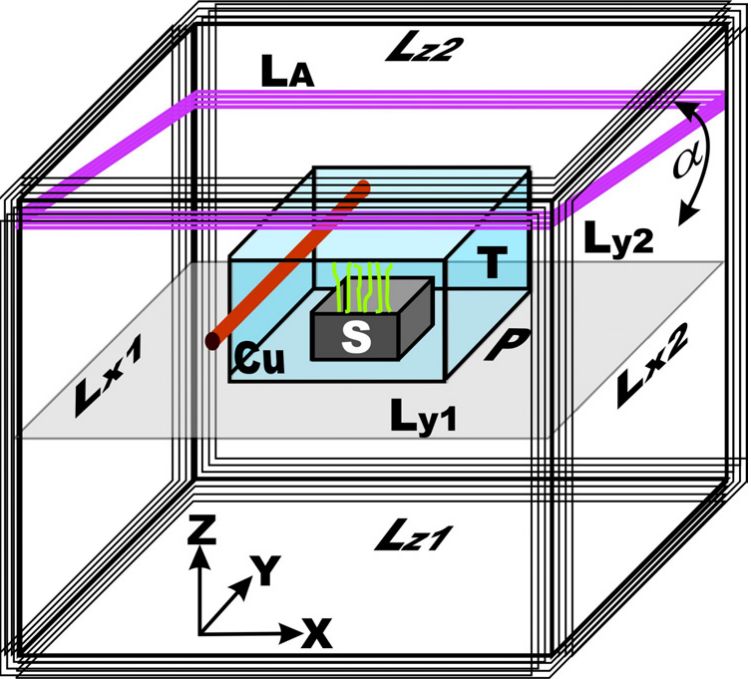
**Experimental facility**. Apparatus for the application of (electro-) magnetic fields. **Lx**1, 2, **Ly**1, 2 and **Lz**1, 2 are coils which enable directed EMF and the compensation of environmental fields. The accessory coil **La **is used in some experiments to generate a magnetic field gradient. For experiments at a fixed field strength, the sample (**S**) is centrally placed, for gradient experiments different locations in Y-direction samples were placed at locations along the middle axis (not shown). The samples were kept in a thermic insulation box (**T**) and temperature is controlled by a small heat exchanger built with copper pipes (**Cu**) driven by a water bath.

A rectangular array of three pairs of square coils (base length of 1 m, L_X1_+L_X2_, L_Y1_+L_Y2_, L_Z1_+L_Z2_) were used in order to enable an independent adjustment of the MF in any direction of space (Fig. 1). With the additional coil L_A_, which can be vertically pivoted by the angle α = 0–20°, a magnetically gradient in Y-direction on the sample plain (P) may be generated with a usable ratio of 1:1.7 for the magnetic fieldstrength. Each single coil consists of 100 turns of coated copper wire (diameter = 0.6 mm).

At a current of about 300 mA, they generated pairwise a static magnetic flux density B_DC _(x,y,z) of 100 μT without any appreciable heat. The samples (S) are centrally placed in a thermal insulation box (T) of styrofoam, temperature is controlled by a small heat exchanger built with copper pipes (Cu) driven by a water bath. A three-axis fluxgate magnetometer (CXM113, Fa. CMT, D-82211 Herrsching) is mounted beneath the sample. Magnetic data are read by a computer which adjusts the desired magnetic field by the coil currents independently via power amplifiers. This unit also allows the superimposition of the signal for the additional alternating magnetic field B_AC_, which is needed to generate ICR conditions, and a partial compensation of possible changes of the local EMF. The magnetic calibration was performed with the Teslameter FM GEO-X (Projekt Elektronik GmbH, Berlin, Germany). The sinusoidal 50 Hz Signal of the AC generator for the alternating field is locked to the power frequency in order to avoid interferences. In order to meet an ICR for Ca^2+ ^at 50 Hz, the sample culture B_DC _is stabilized to 65.8 μT in vertical (z-) direction. B_AC _was parallelly applied with a peak to peak amplitude of 0.5 μT. In the horizontal (x- and y-) direction, the MF was compensated to 0 ± 1 μT for homogenous exposure, and 0 ± 2.5 μT when applying a magnetic gradient. Because of choosing a fixed frequency of 50 Hz for B_AC_, two additional experiments were performed with 5 cultures each simultaneously growing in a field strength gradient in order to verify eq.(1). They were placed at 55, 60, 65.8, 70 and 75 μT (each ± 0.7 μT) vertical magnetic flux density. Control plants were placed outside the apparatus, growing at the local geomagnetic field of about 0.41 μT, but otherwise under the same conditions.

### Investigations on the plants

After 5 days, the boxes were opened under green safety light. 10–30 seedlings were randomly taken from each box and length of shoots, coleoptiles and roots, as well the fresh weight of the whole plants and the detached shoots were determined. Dry weight of leaf and root tissues was obtained after a 24 h desiccation at 105°C in a compartment drier. The coleoptiles from the remaining fresh leaf material were stripped away manually and coleoptile free primary leaves were immediately partitioned to aliquots with known weight for the further investigations: UV-VIS spectroscopy and chromatography of the pigments, studying the greening process by photoconversion tests, and light microscopy.

For the latter, the tissues covered with water were cut with a razor blade after embedding in a styrofoam layer, transferred to an object slide without dying, and inspected with a Zeiss photomicroscope at several magnifications with white as well UV 380 nm fluorescence illumination. Photographs were recorded with a digital microscope camera (model JAI-2040, ProTec, Japan).

For spectroscopy, pigments from the etiolated as well as green leaves were extracted with 90% acetone containing 10 mM NH_4_OH after Rassadina et al. [22]. Spectra were recorded from 400–800 nm with a Perkin Elmer spectrophotometer (Lambda 25), and the concentrations of esterified (Chl a,b) as well of non-esterified pigments (Chlide, PChlide) were determined according to Porra (1991) [25]. Carotenoids were transferred into n-hexane and also quantified by UV-VIS spectroscopy.

The pigment preparations were subsequently concentrated in a rotary evaporator, dried with continuous stream of nitrogen, and stored in a freezer at -18°C for later use. A few of these samples were taken for HPLC analysis on a C-18 reverse-phase column connected to a diode-array spectrophotometer like described in [22]. All gained pigment concentrations were compared by ratios with these of the control plants, which were not exposed to the ICR condition for Ca^2+^.

The photoconversion tests were monitored with a spectrophotometer UV-2401 PC (Shimadzu Duisburg, Germany) which allows analyses of scattering and solid probes by using an integrating sphere after Ulbricht. The absorption spectra were recorded *in vivo *from 600–720 nm several times. For each measurement 10 freshly harvested shoots of medium length were softly fixed on the sample holder that way, so that the part of 5 to 20 mm under the shoot tip matched the optical aperture of the sphere. The endings of the shoots outside the detection area were padded with a moist paper in order to prevent desiccation during the 3 h lasting measurement. A spectrum of the etiolated shoots was recorded, they were then exposed 10 s to white light of about 100.000 l× with a halogen light source (Schott KL 1500 electronic). Immediately afterwards spectra were recorded every minute up to a runtime of 20 min, then the time intervals of measurements were gradually extended until to the end of the experiment, when absorption at 650 nm reached a constant level. Spectra were compiled and the needed datasets were extracted with the software package "Spectacle" (LabControl GmbH). Shibata shift and PChlide regeneration were determined by some obligatory absorption values as ratio functions to initial values [22], having a more or less sigmoidal shape. The Shibata shift S(t) was calculated for the runtime *t *by



and rose from 0 to about 1 for t ~20–25 min. The percentage of PChlide650 regeneration *R*(*t*) was expressed in percent of the immediately converted PChlide after light exposure, and can be obtained by



where the suffix 0 indicates data before, and suffix 1 data directly after light exposure. A0_650 _is the baseline correction factor for 650 nm, which was dependent on sample scattering, and calculated in the first approximation as linear and inverse proportional function of the wavelength. Statistical relevance of all data was checked by the standard deviations and the two sided *Student*-T-test, performed with a spreadsheet software.

## Results

In total 22 experiments with dark growing barley cultures were performed. Standard experiments consisted of two trays, one exposed to the ICR condition for Ca^2+ ^and one control culture. Additionally there were extra experimental series for testing the ICR hypothesis. Two experiments were performed by applying only the static field B_DC _= 65.8 μT with the alternating part B_AC _switched of. Three of the standard experiments were extended by growing a third culture in a permalloy shielding box at an approx. zero (0 ± 1.5 μT) magnetic field. Finally, two experiments with 5 cultures at the same ICR frequency of 50 Hz, but in regions with different static field strengths B_DC_, should help to verify the hypothesized need of coincidence of *f*(B_AC_) = 50 Hz for a static field with the strength B_DC _= 65.8 μT, to match ICR conditions of Ca^2+^.

### Morphology

**Figure 2:**
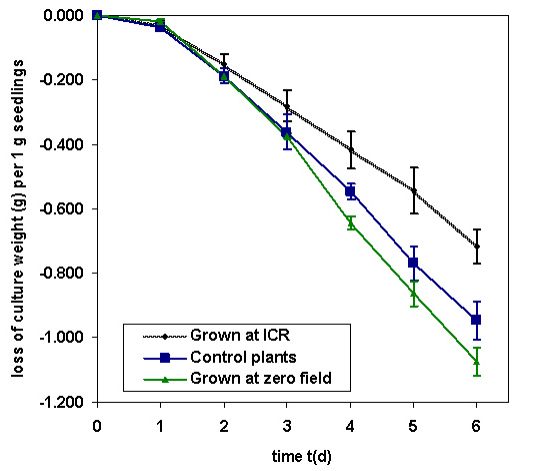
**Relative loss of weight**. Losses of weight, which are due to water evaporation related to the initially used seedling mass of the cultures during the experiments were significantly different for plants etiolated grown under Ca^2+ ^ICR conditions of B_DC _= 65 μT and *f*(B_AC_) = 50 Hz, at zero field conditions, and under unchanged environmental conditions. Standard deviations are shown by bars.

The used plant density of about one seedling per cm^2 ^provided sufficient breeding results and numbers of individuals for subsequent investigations. The water consumption of the cultures was determined by weighing, because it is an indicator for the physiological activity of plants. During 6 days the ICR exposed plants had a significantly lower water loss (3.19 ± 0.23 g) than the control cultures (4.27 ± 0.29 g). The basic water evaporation of 12 g Vermiculite without seedlings under otherwise comparable conditions was 0.39 ± 0.08 g per day, independent of the presence or absence of an EMF. Using this for correction, the averaged net water loss of the cultures exposed to ICR conditions during the first 6 days, is shown in Fig. 2. After the soaking phase at the 1^st ^day, the average daily consumption of the controls (0.16 g·d^-1^) surpasses that of the ICR cultures (0.12 g·d^-1^) by 33%, that of magnetically shielded plants (0.18 g·d^-1^) is even 50% higher. The average percentage dry mass of the ICR exposed cultures was 12% increased for the leaves as well the roots, compared to the control plants.

**Figure 3:**
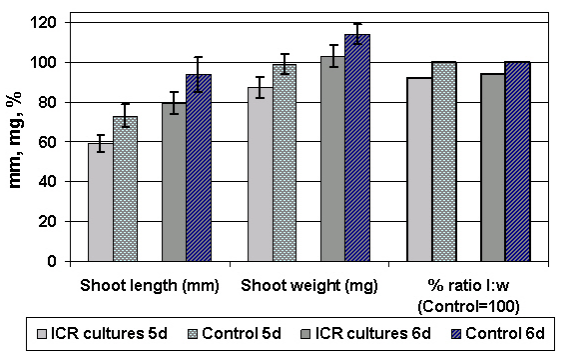
**Length and weight of shoots**. Length and weight of the shoots of barley seedlings etiolated grown under Ca^2+ ^ICR conditions of B_DC _= 65 μT and *f*(B_AC_) = 50 Hz, at days 5 and 6 after germination and of controls grown under the same conditions but in the absence of an EMF. The right two bar groups show the percentage ratios of length to weight (controls normalized to 100%).

**Figure 4:**
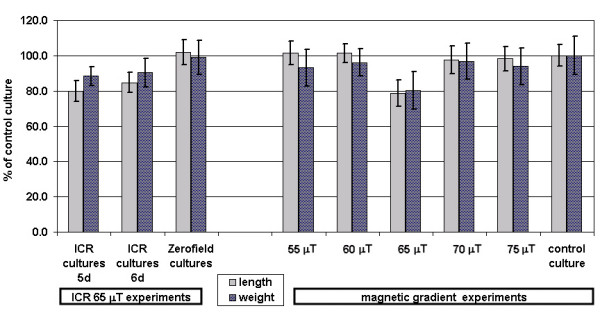
**Shoot growth at different magnetic fields**. The percentage length and weight of the shoots from barley plants etiolated grown in the presence of EMF with respect to those of controls (normalized to 100%) grown under the same conditions but in the absence of an EMF. The left 3 bar groups summarize the data for the experiments with one culture exposed to EMF (B_DC _= 65 μT, *f*(B_AC_) = 50 Hz). The right 6 bar groups show the data from the gradient MF experiments under several field strengths from 55 to 75 μT. A decrease at B_DC _= 65 μT is seen, corresponding to ICR conditions of Ca^2+^.

Several other parameters like growth and mass of the leaves were significantly influenced by the applied EMF. In Fig. 3, the development of length and fresh weight of the barley shoots are displayed. The most important morphological data are compiled in the upper three lines of table 1. On day 5 of incubation the ICR cultures (59.1 ± 4.3 mm) differ from the controls (73.2 ± 5.7 mm) by a factor of 0.805 resp. Δl = 14 mm, which is maintained at day 6, when growth had proceeded for another 15–20 mm. By t-test statistics, a significance level α = 0.0023 is reached for the shoot length. Showing a ratio of 0.884 for the 5^th ^and 0.904 for the 6^th ^day, the mass difference is not as distinctive between the ICR and control cultures, but still significant (α = 0.003). This can be seen, when drawing the length to mass ratios after normalization of the controls to 100% (right bar group in Fig. 3). Plants grown in the shielding box showed with 91.2 ± 4 mm length and 102 ± 10 mg mass, even a slightly increased (~1.5 %) growth compared to the controls, but this difference was not significant (α>0.3). The data of the blind tests were comparable to the controls and did not differ by more than 2.5 %. The effectiveness of the selected ICR condition for Ca^2+ ^could be proved by simultaneously growing several cultures side by side in a gradient field B_DC _of 55–75 μT. Fig 4. shows all shoot length and mass data in percent of the controls (100%). The left groups (3 bar pairs) show again the results for single exposition presented above, including zero field data. The 6 right categories show the corresponding values for cultures grown in a gradient field (55–75 μT), and of control cultures grown outside the gradient. A decrease of about 20% for the plants at 65 μT is here comparable to that of the previous experiments with one ICR and one control culture. The standard deviations are higher because of the lower sample number of these more extensive experiments. Still another observation was made, when manually stripping the coleoptiles from the primary leaf tissue: In the field-exposed cultures, the former adhered more strongly to the latter than in the control plants. Further, coleoptiles had different lengths: Those of the ICR exposed plants were about 12% longer than those of the control plants (45.5 ± 5.2 mm) with a significance of α < 10^-4^.

**Table 1:** Overview of the most important data for growth and pigment contents of barley plants grown under Ca^2+ ^ICR conditions of B_DC _= 65 μT and f = 50 Hz, and of the corresponding control cultures grown in the absence of an EMF. The right column indicates the ratios of the data.

		**ICR exposed cultures**	**Control cultures**	**Ratio: **ICR exposed/control
Averaged daily net water loss (per 1 g seedlings)		**119 ± 24 mg/g**	**158 ± 21 mg/g**	0.75
Shoot length	5 d	**59.1 ± 4.3 mm**	**73.2 ± 5.7 mm**	0.81
	6 d	**79.6 ± 5.4 mm**	**93.9 ± 8.6 mm**	0.85
Coleoptile length (5d)		**50.8 ± 6.5 mm**	**45.5 ± 5.2 mm**	1.12
Shoot fresh weight	5 d	**87.4 ± 5.2 mg**	**99.0 ± 5.1 mg**	0.88
	6 d	**102.9 ± 9.2 mg**	**114.0 ± 8.93 mg**	0.90
percentage dry mass	**leaf**	**7.61 ± 0.29 %**	**6.77 ± 0.35 %**	1.12
	**root**	**6.95 ± 0.33 %**	**6.22 ± 0.24 %**	1.12
Pigment content	5 d: **PChlide**	**7.86 ± 0.89 μg/g**	**10.4 ± 1.23 μg/g**	0.76
	**Carotenoids**	**18.5 ± 1.80 μg/g**	**22.4 ± 3.36 μg/g**	0.85
	^1)^**PChlide/Carot.**	**0.43**	**0.46**	0.92
	6 d: **PChlide**	**8.94 ± 0.88 μg/g**	**11.7 ± 1.84 μg/g**	0.75
	**Carotenoids**	**20.1 ± 1.97 μg/g**	**24.7 ± 1.10 μg/g**	0.82
	^1)^**PChlide/Carot.**	**0.45**	**0.47**	0.94
Pigment ratios of green plants	Chl a+b	706 ± 139 μg/g	990 ± 132 μg/g	0.71
	^1)^Chl a/Chl b	2.42 ± 0.35	2.42 ± 0.29	1.0
	^1)^Chl a+b/Carot.	6.16 ± 0.90	6.95 ± 0.72	0.89

^1) ^ratios

### Photosynthetic pigments

The ratio of spectral PChlide forms *in vivo*, as well as content of the total PChlide of the etiolated barley plants, of Chl *a *and Chl *b *after greening, and the amount of carotenoides where determined. Table 1 gives an overview of the data regarding the pigments accumulated by the barley plants. In many cases they were different for plants grown under electromagnetic conditions of Ca^2+ ^ICR and the control cultures. The PChlide content of 7.86 ± 0.89 μg/g of 5-day-old leaf material from the ICR cultures was significantly lower than the same of the control plants (10.4 ± 1.23 μg/g), with a significance level α < 10^-4^. This trend is observed on the day 6, when plant incubation is continued under unchanged conditions. The average pigment concentrations increased slightly in both cultures during this additional day; the ratios for the ICR plants and the corresponding controls were 0.75 for PChlide and 0.82 for the carotenoids, and were still significantly different (PChlide α = 0.0014, carotenoids α = 0.0013).

**Figure 5:**
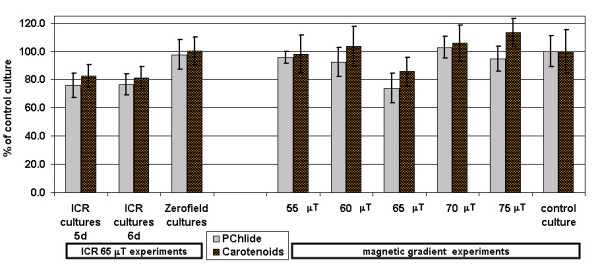
**Formation of photosynthetic pigments**. The percentage amounts of PChlide and Carotenoids from barley plants etiolated grown in the presence of EMF with respect to those of controls (normalized to 100%) grown under the same conditions but in the absence of an EMF. The left 3 bar groups summarize the data for the experiments with one culture exposed to EMF (B_DC _= 65 μT, *f*(B_AC_) = 50 Hz). The right 6 bar groups show the data from the gradient MF experiments under several field strengths from 55 to 75 μT. The pigment concentrations are decreased at B_DC _= 65 μT, corresponding to ICR conditions of Ca^2+^.

Furthermore, the proportion of carotenoids in the total pigment composition was 6–8 % increased for the plants grown under the Ca^2+ ^ICR condition, with a significance of α = 0.015. In Fig. 5 the PChlide and carotenoide concentrations of all experiments with one single culture under ICR, the cultures grown in the shielding box (zero field) and the cultures grown at 5 different locations in a magnetic field gradient (at 55, 60, 65, 70, 75 μT) are shown with respect to an additional control culture outside the EMF exposure apparatus. There is an obvious drop at 65 μT (Ca^2+ ^ICR condition), even though the standard deviations are higher because of the small number of experiments. The significance was α = 0.028 for PChlide and α = 0.047 for the carotenoids.

HPLC analyses of the extracted pigments isolated from etiolated plants shows the relations in detail. They are comparable to the data obtained by the UV-VIS spectroscopy. The PChlide concentration from the ICR exposed samples was the 0.753 fold of this of the controls. Four different carotenoids could by identified by Köst [26] and Panfili et al. [27] with the average percentage composition for the controls: Violaxanthin (18%), Zeaxanthin (27%), Lutein (48%) and β-Carotene (8%). For the ICR cultures, the first two decreased by about 4–5% the latter two increased by the same percentage. However, these data are here still insufficient for a final conclusion.

### Shibata shift and PChlide650 regeneration

**Figure 6:**
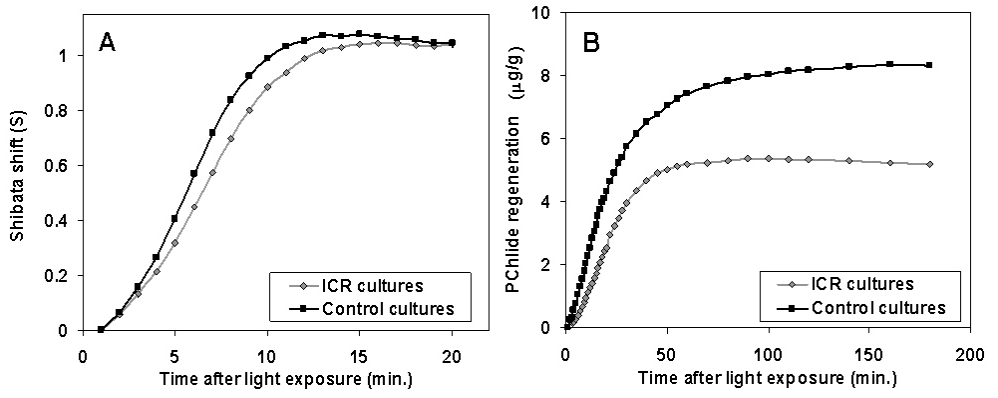
**Shibata shift and PChlide650 regeneration**. **Left: **The Shibata shift indicates the degree of spectral shift from 684 to 672 nm as a normalized function (see eq. (1)). This process seems to be slightly delayed for plants etiolated grown at the Ca^2+ ^ICR (rhomb dotted line) at B_DC _= 65 μT, *f*(B_AC_) = 50 Hz, with respect to those of controls (square dotted line) grown under the same conditions but in the absence of an EMF. **Right: **The regeneration of PChlide650 is calculated by eq. (3) The plants grown at the Ca^2+ ^ICR (rhomb dotted line) have probably a lower activity as controls (square dotted line) grown under the same conditions but in the absence of an EMF.

The etiolated leaves were irradiated with a tungsten lamp (1500 W/m^2^) for 10 s, for sufficient light saturation to reduce photoactive PChlide. UV-VIS Spectra were automatically recorded from the leaves *in vivo *for 3 h as described above. It is known, that illumination of etiolated plants causes besides PChlide photoreduction the disaggregation of prolamellar bodies and the relocalization of PChlide-oxidoreductase (POR) and Chl synthase from prolamellar bodies to prothylakoids, which are observed together with the Shibata shift and, thereby, Chlide esterification. Shibata shift is seen by a shift of chlorophyllide (Chlide) absorption maximum from 684 to 672 nm during 20–30 min. after light exposure. Data are normalized, as above, thereby they are largely independent of the initial pigment concentration. Therefore, these data were averaged from the single experiments, resulting in the two graphs shown in fig. 6A (left) for 5d old plants. The Shibata shift of the ICR plants is slightly delayed compared to the controls, with the largest difference of 0.13 after 7 min., it then converges to equal values at the end of the process at approx. 20 min after the light irradiation.

The regeneration of the photoactive PChlide650 in etiolated seedlings after light irradiation is closely connected with *de novo *synthesis of PChlide, availability of NADPH, and POR to form ternary complex with pigment. It begins with a 15–20 min delay, and is also evaluated by normalization (PChlide content before irradiation = 100%). The ICR plants reach a regeneration of 84.0%, the controls 96.5% after 180 min, with a ratio of 0.89 averaged about any of the 40 corresponding single recordings during this time, and final significance level for the differences of α = 0.0005. The difference appears still more clear, if the percentage rates are related to the amounts of involved PChlide. In fig. 6B (right) the time-dependent PChlide concentrations related to 1 g fresh leaf material (without coleoptiles) are displayed for the observation time. Here the PChlide content of leaves grown under Ca^2+ ^ICR condition reach only 63% of the control samples.

The ratio between the absorption intensity at 650 and 630 nm in spectra of etiolated leaves characterizes the relative amount of photoactive and non-photoactive PChlide forms. It seems that ICR conditions lead to relative rise of photoactive PChlide650 form in etiolated barley. The ratio between the absorption intensity of PChlide650 and PChlide630 in ICR leaves is 1.49 ± 0.085 and in control ones is 1.32 ± 0.093, but with a low significance (α = 0.2).

### Further development of the adult plants

The barley plants not used for the aforementioned investigations (approx. 50%) were grown after the 5^th ^day under natural day-night conditions in an experimental greenhouse, without any artificial EMF exposition. After one week (12 d total cultivation time) the pigment content was determined again by UV-VIS spectroscopy. A total Chl (a+b) concentration of 706 ± 139 μg/g in the ICR plants corresponds to 71.4% of control value. Remarkably, the ratios of Chl *a *to Chl *b *did not differ between ICR plants and controls, they were in any case 2.42.

**Figure 7:**
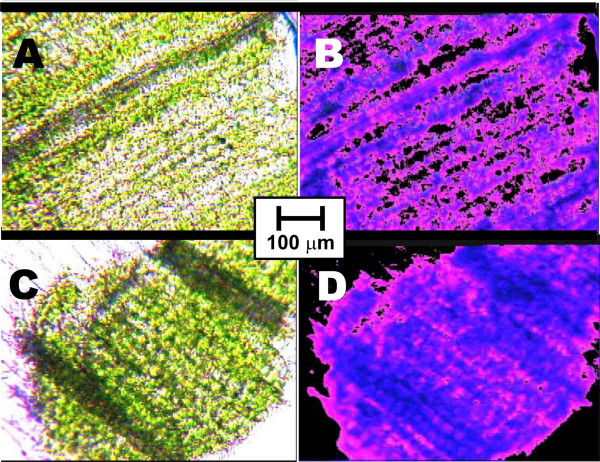
**Microphotos of leaf tissues**. Plants were grown initially 5d etiolated, then 7d in the light. Microphotos A and B belong to sections of plants additionally exposed to Ca^2+ ^ICR condition (B_DC _= 65 μT, *f*(B_AC_) = 50 Hz) during the 5d etiolation phase, while C and D are from comparable preparations of controls grown under the same conditions, but without Ca^2+ ^ICR. A and C are taken under tungsten light, B and D under fluorescent illumination (380 nm). The plants grown at the Ca^2+ ^ICR seemingly show a lower chloroplast density and an increased (red) fluorescence caused by free chlorophylls.

**Figure 8:**
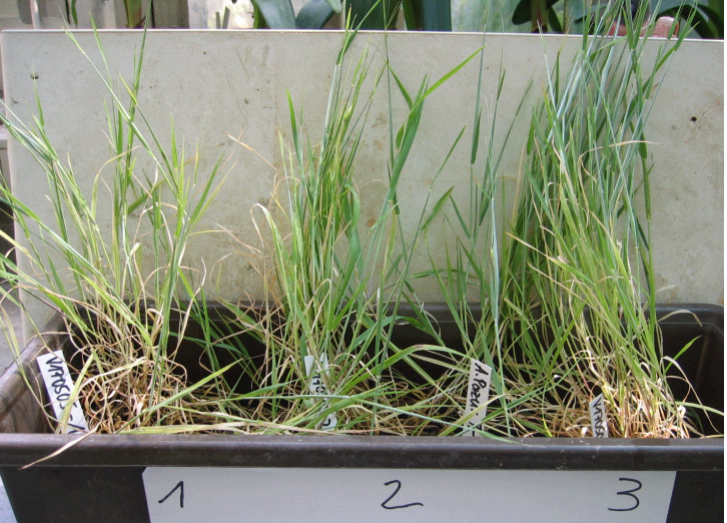
**Long-term development of the light-grown barley plants after Ca^2+ ^ICR exposure**. Barley plants after 10 weeks greenhouse cultivation (potted on soil in one tray, daylight with natural day-night cycle, moderate temperature around 21°C). Plants were initially grown 5 d in the dark: (1, left) exposed to Ca^2+ ^ICR condition (B_DC _= 65 μT, *f*(B_AC_) = 50 Hz), (2, middle) in a shielding box, (3, right) was a control culture of plants grown under the same conditions but in the absence of Ca^2+ ^ICR. Even after this long time the retardation of the Ca^2+ ^ICR culture (1) was still visible.

Additionally preparations of leaf tissue were viewed by a microscope: Fig. 7 displays photomicrographs taken from longitudinal sections of ICR plant leaves (A) and controls (C) under tungsten light, with the common scale shown in the center. Photographs B and D in the right show the same preparations under fluorescent illumination (380 nm). (A) indicates more loose distributed chloroplasts than the controls (C), also the fluorescence caused by free chlorophylls (B) is increased in comparison with the control sample (D). A stronger fluorescence of Chl could be a measure for possible impairments of the photosynthetic apparatus in the plants exposed to the electromagnetic condition of Ca^2+ ^ICR. With the intention to raise some of these plants to seed production, observations were continued for several weeks. Fig. 8 shows cultures after 10 weeks. All plants were grown etiolated for the first 5 days: The left (1) were exposed to Ca^2+ ^ICR, the middle (2) grew in a shielding box for this time, the right (3) was a control culture. Even after this long time the retardation of the ICR culture (1) was still obvious, visible by lower growth, and common drought symptoms like more yellowish leaves.

## Discussion

Judged from several parameters, growth of barley seedlings is diminished by a combination of a static and an alternating magnetic field, which corresponds the ICR of Ca^2+^. No ferrimagnetic particles were found up to now in barley plants, which could be directly influenced by MF. Also a radical pair effect e.g. on charge separation during photosynthesis as primary magnetoreceptor is improbable in the dark. There are also hardly other environmental stress factors, which could force radical generation. Furthermore, radical pair reactions should not show a resonant response like the one seen here, in the gradient experiments, which showed maximum effects for B_DC _= 65 μT and *f*(B_AC_) = 50 Hz, directly relating the ICR equation for Ca^2+ ^(1). Finally the radical pair mechanism should not be influenced by as low field strengths as were applied here. Seemingly the visible appearance as well as regulative processes of the plants are similarly pertained by the applied EMF, whereby there are possibly morphological manifestations lasting for extended times beyond the phase of exposition to Ca^2+ ^ICR.

Several theoretical models try to explain the ICR effect and its possible biological implications. The model of Binhi [28] is based on an interference mechanism of quantum states of ions within protein cavities. Different types of orbits are considered, and a formula has been developed for the magnetic field-dependent part of the dissociation probability of an ion-protein, in order to explain the resonance behavior of the system. More recent experiments could show similar effects in the absence of proteins or interface building structures like lipid membranes: A resonant EMF effect could already be shown by the change of the conductivity of amino acid solutions, if they were exposed to the respective ICR condition [29,30]. Such results challenged additional theoretical models. The major difficulty in rationalizing the effects as a result of ICR is the requirement of undisturbed *Larmor *precession of charged particles in a condensed phase, viz. aqueous environment, at ambient temperatures. It has been based on the quantum electrodynamical properties of water, which however are still only poorly studied. Del Giudice et al. [31] propose a two phase state of water with regions providing quantum coherence, and thereby a decoupling factor against the surrounding thermodynamical equilibrium. Already earlier Novikov et al. [32] suggested a self-organizing mechanism for larger ion assemblies and substantiated this theory by applying motion equations for basic forces of only ~10^-21 ^– 10^-25 ^N for the *Lorentz*ean force on an ion from an external magnetic field. Further, the coupling of electron spin states for comparably long distances and time windows could provide a resonant EMF effect for radical pairs [33], representing a special case of the common radical pair effect and enhancing its sensitivity for EMF by orders of magnitude. As mentioned above, it is very difficult to explain the EMF effects found in the present study by the established mechanisms for biological magnetoreception: ferrimagnetic particles or the radical pair mechanism. Two questions remain: The existence of a predictable resonance phenomenon, and the high sensitivity to EMF of only some 10–100 nT., which must be overlaid to a static magnetic field at most in the order of the geomagnetism (≤100 μT).

Ca^2+ ^is an important factor for the bioregulation in plants. So it is surely a useful approach to postulate a modulation of the Ca^2+ ^supply of the plants by the electromagnetic impact, also proposed by Huang et al. [34], and subsequently to consider specific aspects of Ca^2+ ^regulation in plants, compiled by Medvedev [20]. It is obvious, that effects should preferentially occur at locations with increased Ca^2+^-concentrations and on Ca^2+^-mediated transport systems. The Ca^2+^-concentration in cell walls and vacuoles is up to 100 times higher than in the cytosol, the cell membranes provide sharp gradients of Ca^2+ ^concentration, along with a electrochemical potential, which is widely modified by the activity of membrane channels. Phytochrome A activity and circadian regulation are driven by Ca^2+ ^oscillations, which are discussed as part of the "biological clock" of plants [35]. The influence on cellular structure, a subsequent smaller growth, damages on the photosynthetic apparatus, and decreased Chl and carotenoid concentrations caused by calcium and magnesium deficiency are described for barley plants by Klyachenko [36]. These effects were qualitative similar, even though less pronounced, in the current experiments, which would be consistent with an influence of the used MF and EMF on the Ca^2+ ^regulation.

Lower PChlide content in etiolated ICR leaves could be connected with reduced amount of plastids per cell as well reduced plastid size, or intraplastid membrane quantity – parameters genetically programmed for each plant cell species. The ratio between the absorption intensity of PChlide650 and PChlide630 in Ca^2+ ^ICR exposed leaves belongs rather to the unexpected result. It is known that many treatments, like exogenous phytohormones, high temperature, irradiation with far-red light and some mutations induce the enhancement of photononactive PChlide. Reduced rate of the PChlide resynthesis as well as its end level under ICR conditions could be results on limited biosynthesis of PChlide, NADPH or POR.

As rate of Shibata shift and Chlide esterification is practically equal [22], it is obviously, that applied treatment has not significant influence on the Chl formation in short illuminated etiolated leaves. Lower Chl content and retarded plant development during prolonged growing of ICR barley point to pleiotropic effects of used EMF, connected probably with realization of genetic program and energy metabolism. More detail experiments are needed to observe what of this is disrupted under ICR conditions.

Grossly speaking, all processes depend from the intracellular water supply, and just here the data indicate an increased drought stress for the ICR exposed plants. Considering the data shown in fig. 2, already a decreased water uptake could be the matter more than an increased evaporation. In [37], wheat plants were investigated after applied drought stress, they indicated an enhanced oxidative damage to photosynthetic pigments, proteins and lipids. Essential enzymes like superoxide dismutase, catalase and glutathione reductase showed a decreased activity. Lastly Le Lay et al. [38] report desiccation-induced delays and reductions of the chlorophyllide transformation in etiolated barley leaves, similar to our observations on the ICR exposed plants. Because also a temporary water deficit may cause irreversible damages, the observed adverse manifestations on the plants long time after finishing ICR exposure can well be rationalized.

## Conclusion

In summary, an increased desiccation of the barley plants exposed to the ICR condition for Ca^2+ ^could explain the different effects observed. This raises the question, if a decreased water supply is primarily caused by active regulation processes or by the properties of the electrolytes, e.g. an altered availability of Ca^2+ ^induced by the electromagnetic field. Also, a change of the viscosity, caused by the hypothesized quantum electrodynamical properties of water, is conceivable, that hinders water transport in the plants. It will be expected, that a diminished intracellular supply of water and electrolytes caused by certain combinations of weak MF and EMF could also occur in other organisms, above all if the ICR effect would be mediated by properties of the water-ion environment itself, without the need of distinct arrangements of specific proteins and lipid membranes. Combinations of weak MF and EMF leading to resonance condition for Ca^2+ ^are not uncommon: The geomagnetic field would serve as the static MF component, the overlaying EMF may originate from natural phenomena, e.g. atmospheric processes, as well from man made sources, like electric supplies. Kaiser tried already in 1996 to compute a comprehensive oscillator theory for resonant EMF effects in biological systems [39]. Considering possible consequences for public health care as well as common ecological relations, more work is needed to clarify the basics of biological effects by weak electromagnetic fields.

## List of abbreviations

**Chl a/b: **Chlorophyll a/b

**EMF: **(low frequency) Electromagnetic field

**HPLC: **High performance liquid chromatography

**ICR: **Ion cyclotron resonance

**MF: **Magnetic field

**(P)Chlide: **(Proto-)Chlorophyllide

**POR: **PChlide-oxidoreductase

## Authors' contributions

The authors carried out the experiments and drafted the manuscript.
